# Endocannabinoid receptor 2 is a potential biomarker and therapeutic target for the lysosomal storage disorders

**DOI:** 10.1002/jimd.12813

**Published:** 2024-11-21

**Authors:** Calogera M. Simonaro, Makiko Yasuda, Edward H. Schuchman

**Affiliations:** ^1^ Department of Genetics & Genomic Sciences Icahn School of Medicine at Mount Sinai New York New York USA

**Keywords:** biomarkers, endocannabinoids, lysosomal storage disorders, therapeutic target

## Abstract

Herein, we studied the expression of endocannabinoid receptor 2 (CB2R), a known inflammation mediator, in several lysosomal storage disorder (LSD) animal models and evaluated it as a potential biomarker and therapeutic target for these diseases. CB2R was highly elevated in the plasma of Farber disease and mucopolysaccharidosis (MPS) type IIIA mice, followed by Fabry disease and MPS type I mice. Mice with acid sphingomyelinase‐deficient Niemann‐Pick disease (ASMD) and rats with MPS type VI exhibited little or no plasma CB2R elevation. High‐level expression of CB2R was also observed in tissues of Farber and MPS IIIA mice. Treatment of MPS IIIIA patient cells with CB2R agonists led to a reduction of CB2R and monocyte chemoattractant protein‐1 (MCP‐1), a chemotactic factor that is elevated in this LSD. Treatment of MPS IIIA mice with one of these agonists (JWH133) led to a reduction of plasma and tissue CB2R and MCP‐1, a reduction of glial fibrillary acidic protein (GFAP) in the brain, and an improvement in hanging test performance. JWH133 treatment of Farber disease mice also led to a reduction of MCP‐1 in tissues and plasma, and treatment of these mice by enzyme replacement therapy (ERT) led to a reduction of plasma CB2R, indicating its potential to monitor treatment response. Overall, these findings suggest that CB2R should be further examined as a potential therapeutic target for the LSDs and may also be a useful biomarker to monitor the impact of therapies.

## 1 INTRODUCTION

The lysosomal storage disorders (LSDs) are a group of over 60 inherited metabolic diseases.^1^ Most are caused by deficiencies of degradative lysosomal enzymes, resulting in the accumulation of macromolecules and lysosomal dysfunction. These abnormalities ultimately result in many common, downstream pathologies, including oxidative stress, inflammation, and cell death, leading to organ dysfunction, reduced quality‐of‐life, and/or premature death in patients.^2^

Treatment of the LSDs is generally accomplished by replacement of the defective enzyme, either by organ transplantation, enzyme replacement therapy (ERT), or gene therapy.^3^, ^4^ ERT is the current “gold standard” for these diseases and Food and Drug Administration (FDA)‐approved therapies are available for 11 LSDs. Several small‐molecule therapies are also approved for individual diseases, as are therapies that target downstream mechanisms. However, despite the success of these therapies, the majority of LSDs lack disease‐modifying treatments, and even for those where treatments exist, unmet medical needs persist for many patients.^5^, ^6^

Another current challenge in the LSD field is the identification of biomarkers that can be easily measured in patients and are directly linked to the underlying metabolic defects.^7^, ^8^ Such biomarkers are important for monitoring the disease state and the impact of treatments. This is particularly important in the emerging era of newborn screening, where patients may be diagnosed by enzyme or DNA testing prior to their clinical presentation, leading to difficult and often uncertain decisions regarding their care and treatment.^9^ LSD animal models have been essential for biomarker discovery and validation, as well as for the development and evaluation of new therapies.^10^, ^11^

The endocannabinoid system is comprised of several bioactive lipids that interact with two cannabinoid (CB) receptors, CB1R and CB2R.^12^, ^13^ CB1R is expressed primarily in the brain and to a lesser extent in peripheral tissues, while CB2R is found in circulating immune cells, the spleen, and on other macrophage‐derived cells (e.g., Kupffer cells in the liver). Unlike the widespread expression of CB1R in the CNS, the expression of CB2R in the brain is generally low and restricted to the brainstem and hippocampal neurons. However, its expression is highly elevated following brain injury and/or inflammation, where it is primarily seen in the microglia. Engagement of CB1R and CB2R by endocannabinoid lipid mediators, which themselves are regulated by a series of synthetic and degradative enzymes, leads to downstream signaling events that contribute to cell function. In the case of CB2R, this includes anti‐inflammatory effects, blood–brain barrier protection, and other outcomes that are highly relevant to the LSDs.^14^

In this study, we assessed the expression of CB2R in several LSD animal models and evaluated it as a potential biomarker and therapeutic target for these diseases.

## 2 MATERIALS AND METHODS

### 2.1 Animals

Breeding colonies of mice with acid sphingomyelinase‐deficient Niemann‐Pick disease (ASMD),^15^ Fabry disease,^16^ Farber disease,^17^ mucopolysaccharidosis (MPS) type I,^18^ and MPS type IIIA,^19^ and a rat colony of MPS type VI,^20^ were established as described previously. Homozygous/hemizygous (in the case of Fabry disease due to its X‐linked inheritance) and wild‐type (WT) male and female animals were detected by genotyping using tail clip DNA. All experiments were performed at the Icahn School of Medicine at Mount Sinai under protocols (#'s 08‐0108, 98‐0089, 2013‐1407, 202000236 and 202100005) approved by the Institutional Animal Care and Use Committee, and adhered to the NIH guidelines for the use of experimental animals. Animals were fed ad libitum food and water, and euthanasia was performed by ketamine/xylazine injections followed by cervical dislocation according to NIH guidelines.

### 2.2 Antibodies and other reagents

An anti‐CB2R antibody that cross‐reacted with both mouse and rat CB2R was obtained from ABclonal (Woburn, MA) (#A1762). Anti‐mouse monocyte chemoattractant protein‐1 (MCP‐1) and glial fibrillary acidic protein (GFAP) antibodies were obtained from Abcam (Walton, MA) (#'s ab25124 and ab7260). The CB2R agonists, JWH133 and PM226, were obtained from Caymen Chemicals (Ann Arbor, MI) and R&D Systems (Minneapolis, MN), respectively (#'s 10 005 428 and 6220). Mouse‐specific CB2R and MCP‐1 enzyme‐linked immunosorbent assay (ELISA) kits were obtained from Lifespan Biosciences Inc. (Seattle, WA) and R&D Systems Inc. (Minneapolis, MN), respectively (#'s LS‐F38480‐1 and MJE008). All other standard reagents were obtained from ThermoFisher Scientific (Waltham, MA) or other commercial sources.

### 2.3 Determination of CB2R in plasma and tissues

Farber disease, Fabry disease, ASMD, and MPS I and IIIA mice were aged (2, 8, 5, 8, and 8 months of age, respectively). Age‐ and gender‐matched WT mice were obtained from within the colonies as controls. MPS VI and WT rats were also aged to 8 months. At the time of euthanasia, blood was obtained by cardiac puncture, and plasma was collected in heparin tubes. Tissues were also harvested, fixed in 4% formalin, and paraffin‐embedded, and 5‐micron sections were prepared using a vibratome for immunohistochemical (IHC) analysis. CB2R levels in plasma from each animal model (*n* = 8/group) were determined by ELISA in triplicate according to the manufacturer's instructions. The results were compared to age‐matched WT animals. Since there was little variation among the WT animals from the individual mouse colonies, the WT data was combined (*n* = 38) to obtain greater statistical power. For the IHC analysis, the sections were washed with phosphate buffered saline (PBS) and preincubated with 3% H_2_O_2_ for 15 min to remove endogenous peroxidase activity. To minimize nonspecific immunostaining, the sections were incubated for 60 min with a blocking solution of 2% BSA in PBS. The sections were reacted overnight at 4°C with the primary antibody against CB2R (dilution 1:20) in a blocking solution containing 0.3% Triton X‐100. Following several washes in PBS for 30 min, the sections were reacted with biotin‐conjugated anti‐rabbit second antibody for 60 min at room temperature. The immunoreaction was completed using avidin‐biotin‐peroxidase and the Vectastain ABC kit (Vector Laboratories, Burlingame, CA). The color reaction was visualized using the diaminobenzidine (DAB) substrate, enhanced with 2% ammonium nickel (II), and analyzed using a Nikon Eclipse microscope. For each animal/tissue studied, at least 5 sections were stained and visualized.

### 2.4 Determination of MCP‐1 and GFAP in plasma and/or tissues

Plasma levels of MCP‐1 were determined in triplicate from aged Farber and MPS IIIA mice and age‐matched WT controls by ELISA according to the manufacturer's instructions. IHC detection of MCP‐1 and GFAP in tissue sections were determined as described above, except for the use of MCP‐1 (dilution 1:200) or GFAP (dilution 1:1000) primary antibodies.

### 2.5 Evaluation of CB2R agonists in cells

An MPS IIIA patient skin fibroblast cell line was obtained from the Coriell Institute for Medical Research Cell Repository (Camden, NJ). The cells were grown in Eagles MEM media containing 10% fetal bovine serum (FBS). Upon reaching ~70% confluency, the media was removed and JWH133 or PM266 were added to fresh FBS‐free media at a final concentration of 3 mM, and the cells were grown for an additional 72 or 96 h. The media was then collected, and MCP‐1 levels were determined by ELISA as described above for plasma. Duplicate cultures were prepared, and the MCP‐1 ELISA assays were performed in triplicate on each culture. Control cultures were grown using 0.1% DMSO, representing the final concentration of this solvent in the media.

### 2.6 Evaluation of JWH133 in MPS IIIA and Farber mice

Three‐month‐old MPS IIIA mice received once‐weekly IP injections of JWH133 (10 mg/kg) for 7 months, and a control group received injections of 0.1% DMSO alone (*n* = 8/group). At the end of the treatment period, the mice were euthanized, and the plasma and tissues were analyzed by ELISA (CB2R or MCP‐1) or IHC (GFAP) as described above. A hanging test was used to assess motor strength and coordination as previously described.^21^ 2.5‐week‐old Farber disease mice were injected IP with JWH133 (10 mg/kg; *n* = 8) or vehicle alone (*n* = 10) once per week for 6 weeks. At the end of the study, the mice were euthanized, and MCP‐1 was detected in plasma by ELISA or in the liver by IHC as described above.

### 2.7 Evaluation of CB2R following ERT in Farber mice

Recombinant human acid ceramidase (rhAC) was prepared from an overexpressing Chinese hamster ovary cell line as previously described.^22^ For the ERT experiments, 2.5‐week‐old Farber disease mice were injected intraperitoneal (IP) with rhAC once per week at a dose of 10 mg/kg or with PBS alone (*n* = 8/group). After 6 injections, the mice were euthanized, and the plasma and liver were collected for CB2R analysis. ELISA and IHC detection of CB2R were performed as described above.

### 2.8 Statistical evaluation

For two‐group comparisons, the Mann–Whitney *U*‐test for non‐parametric data or a two‐sample Student's *t*‐test for data with parametric distribution was used. For multiple comparisons, data with a normal distribution were assessed using a one‐way ANOVA followed by a Tukey post hoc test. The statistical significance of non‐parametric data was determined by the Kruskal–Wallis test for all experimental groups. *p*‐values (*p*) < 0.05 were considered significant and the values are indicated on the individual figures. GraphPad Prism 6.0 software (GraphPad Software, La Jolla, CA, USA) was used for all statistical analysis.

## 3 RESULTS

### 3.1 CB2R levels in LSD animal models

**FIGURE 1:**
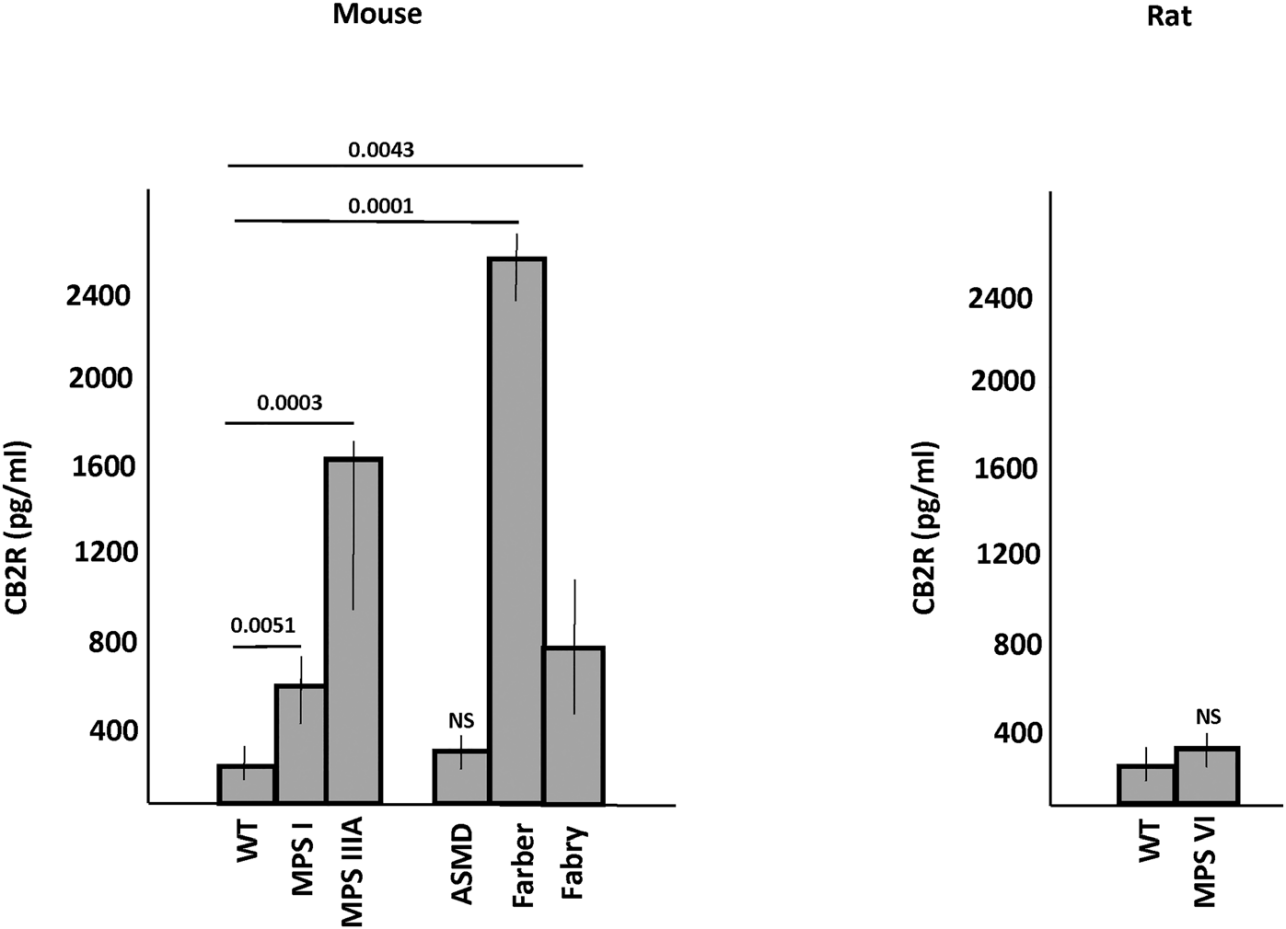
CB2R plasma levels in lysosomal storage disorder (LSD) animal models. Plasma was collected from aged LSD mice and rats and CB2R levels were measured by ELISA. At the time of the analysis the mucopolysaccharidosis (MPS) I mice were 8 months, MPS IIIA mice 8 months, ASMD (ASMKO mice) 5 months, Farber mice 2 months, and Fabry mice 8 months. The MPS VI rats were 8 months of age. Results were compared to wild‐type (WT) animals derived from within the individual colonies. Since there was little variation among the WT mice from different colonies, genders and ages, these data were combined to generate more statistical power (*n* = 38 for WT and 8 animals/group for each of the disease models). Farber, MPS IIIA, Fabry and MPS I mice exhibited significant differences when compared to WT (*p* values are indicated). Bar heights indicate the mean values from three independent experiments. NS = non‐significant (*p* > 0.05).

**FIGURE 2:**
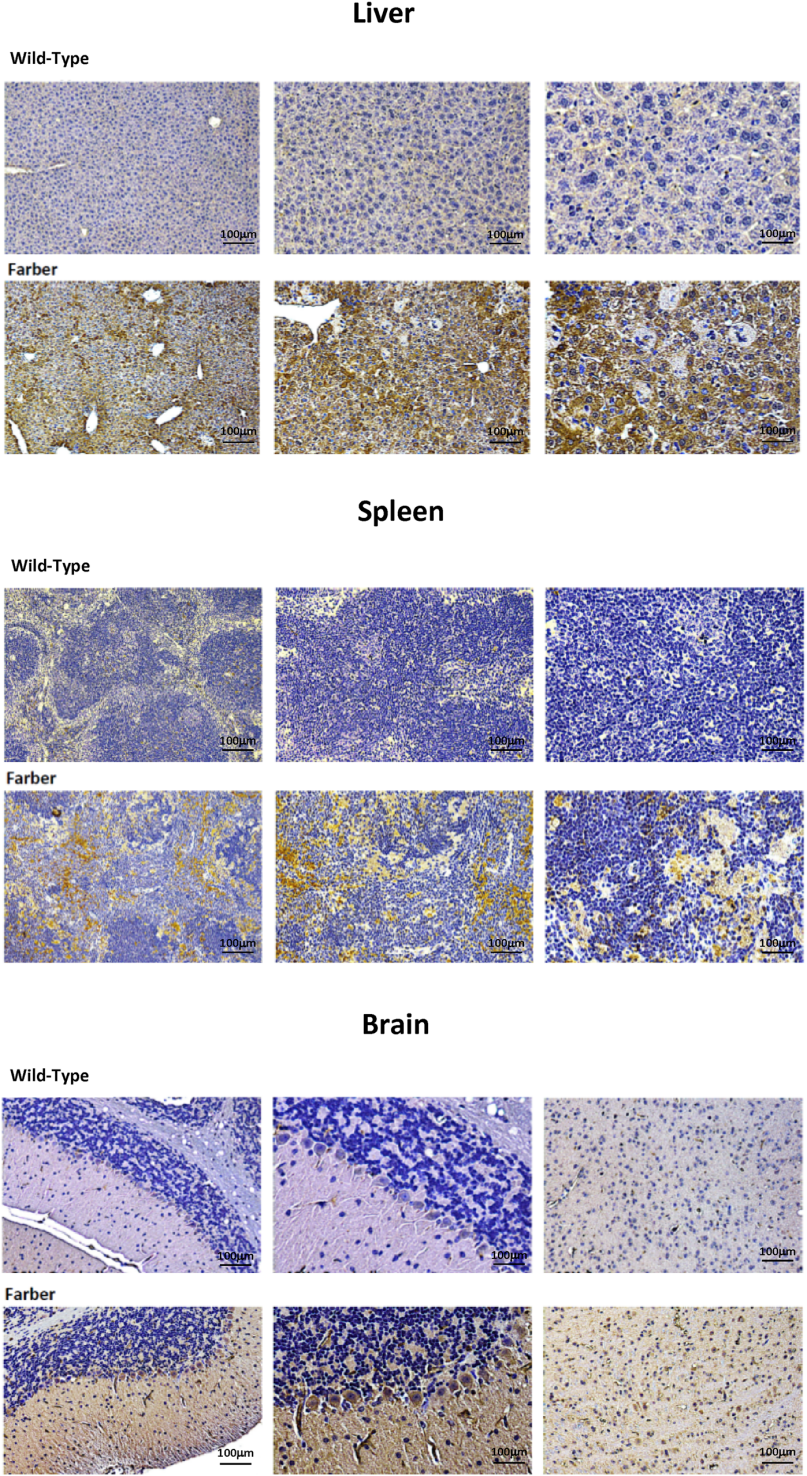
CB2R expression in Farber disease mouse tissues. CB2R was detected in tissues of 2‐month‐old Farber and wild‐type (WT) mice by immunohistochemical (IHC). Six mice/group were analyzed and representative images are shown (a minimum of 5 sections were analyzed/mouse). Brown staining indicates CB2R.

**FIGURE 3:**
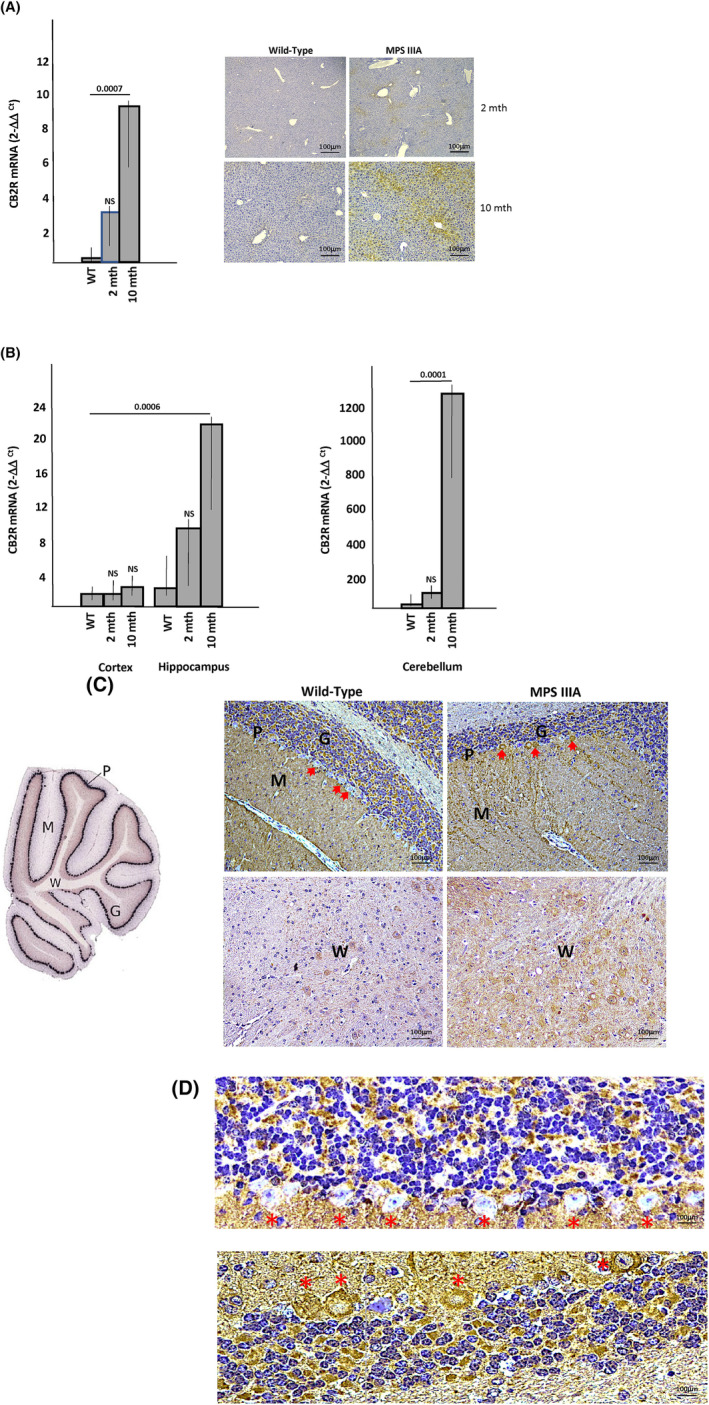
CB2R expression in MPSIIIA mouse tissues. (A) mRNA was prepared from liver homogenates of 2‐ and 10‐month‐old mucopolysaccharidosis (MPS) IIIA mice and 10‐month‐old WT mice (*n* = 10/group). qPCR analysis was then performed to assess the levels of *Cb2r* mRNA. A significant elevation was noted at 10 months of age (*p* value is indicated). Bar heights indicate the mean values from three independent experiments. NS = non‐significant. Representative images of liver sections from 2‐ and 10‐month‐old WT and MPS IIIA mice stained by IHC for CB2R is shown to the right (6 mice/group and a minimum of 5 sections/mouse were analyzed). Brown staining indicates CB2R. (B) mRNA was prepared from brain homogenates of 2‐ and 10‐month‐old MPS IIIA mice and 10‐month‐old WT mice (*n* = 10/group). qPCR analysis was then performed to assess the levels of *Cb2r* mRNA. By 10 months of age significant differences were observed in the hippocampus and cerebellum, but not the cortex (*p* values are indicated). Bar heights indicate the mean values from three independent experiments. NS, non‐significant (*p* > 0.05). (C) Immunohistochemical (IHC) detection of CB2R in the cerebellum of MPS IIIA mice. Brown staining indicates CB2R. For reference an image of the cerebellum is shown to the left with the regions indicated. Red stars indicate Purkinje cell neurons, which are shown at higher magnification in (D). P = Purkinje cell neurons, M = molecular region, G = granule layer, and W = white matter. Note the brown staining in Purkinje cells of MPS IIIA mice compared to WT.

Plasma from WT and age‐matched MPS I, MPS IIIA, ASMD, Farber disease, and Fabry disease mice were analyzed for CB2R expression by ELISA. Aged‐matched WT and MPS VI rat plasma also were studied. As shown in Figure 1, CB2R levels were significantly elevated in Farber, MPS IIIA, MPS I, and Fabry disease mouse plasma compared to WT. There was no significant elevation of CB2R in ASMD mice or MPS VI rats. We next used IHC to evaluate CB2R expression in tissues from the Farber disease mice, which had the highest plasma levels among the LSD models studied. As shown in Figure 2, CB2R was highly elevated in the liver, spleen, and brain (cerebellum) of these animals. Immunohistochemistry and qPCR analyses also revealed elevated CB2R/*Cb2r* expression in the liver from MPS IIIA mice, evident by 2 months of age and highly significant by 10 months (Figure 3A). *Cb2r* gene expression was also highly elevated in the hippocampus and cerebellum of MPS IIIA mice by 10 months of age, although not in the cortex (Figure 3B). Within the cerebellum, CB2R expression was particularly evident in the Purkinje cell neurons (Figure 3C,D). Overall, these results showed that CB2R was elevated in plasma and tissues of several LSD animal models, indicating its potential as a biomarker of disease progression and pathology.

### 3.2 Evaluation of CB2R as an indicator of treatment response

**FIGURE 4:**
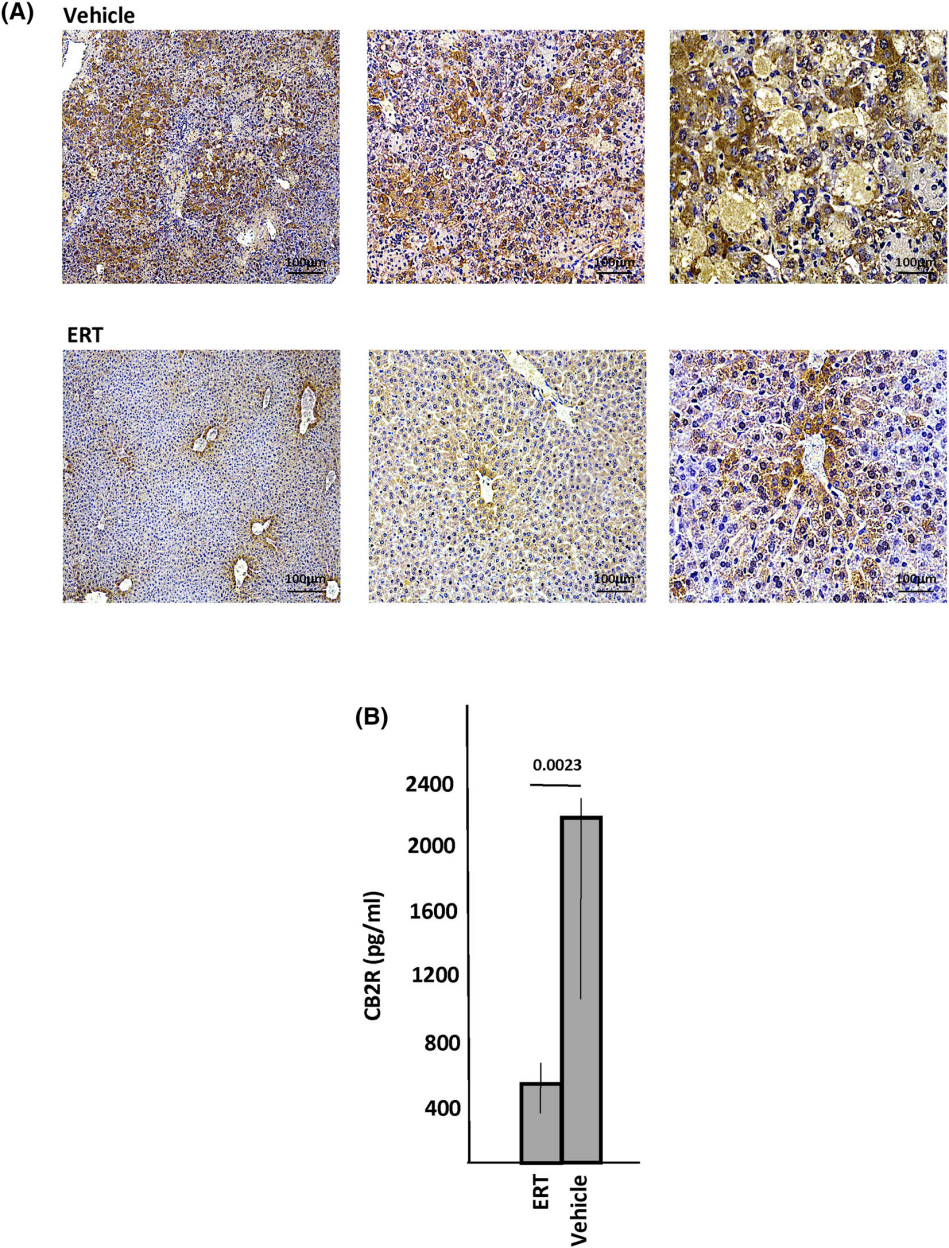
CB2R levels in Farber disease mice following enzyme replacement therapy (ERT). Ten 2.5‐week‐old Farber disease mice were treated once weekly with recombinant acid ceramidase by intraperitoneal injection at a dose of 10 mg/kg. After 6 injections the 8.5‐week‐old mice were euthanized and their livers collected and processed for immunohistochemical (IHC) detection of CB2R (A) or detection of CB2R in plasma by ELISA (B). Representative images are shown (a minimum of 5 sections/mouse were analyzed). Brown staining indicates CB2R. *P* values are shown in (B), and bar heights indicate the mean values from three independent experiments.

We next treated Farber disease mice by ERT using recombinant acid ceramidase.^23^ Previous studies have shown that this treatment led to a reduction of ceramide and several inflammation markers, leading to improvement in cellular pathology and several clinical parameters. After 7 weeks of ERT treatment (once weekly, 10 mg/kg), the Farber mice were euthanized, and their plasma and liver were analyzed by ELISA or IHC for CB2R expression. As shown in Figure 4A, ERT led to a significant reduction of CB2R expression in the liver, corresponding to the known impact of this treatment on ceramide storage and inflammation.^23^ Similar results were observed in the plasma (Figure 4B). The brain was not analyzed since ERT does not impact the CNS.

### 3.3 Evaluation of CB2R as a therapeutic target

**FIGURE 5:**
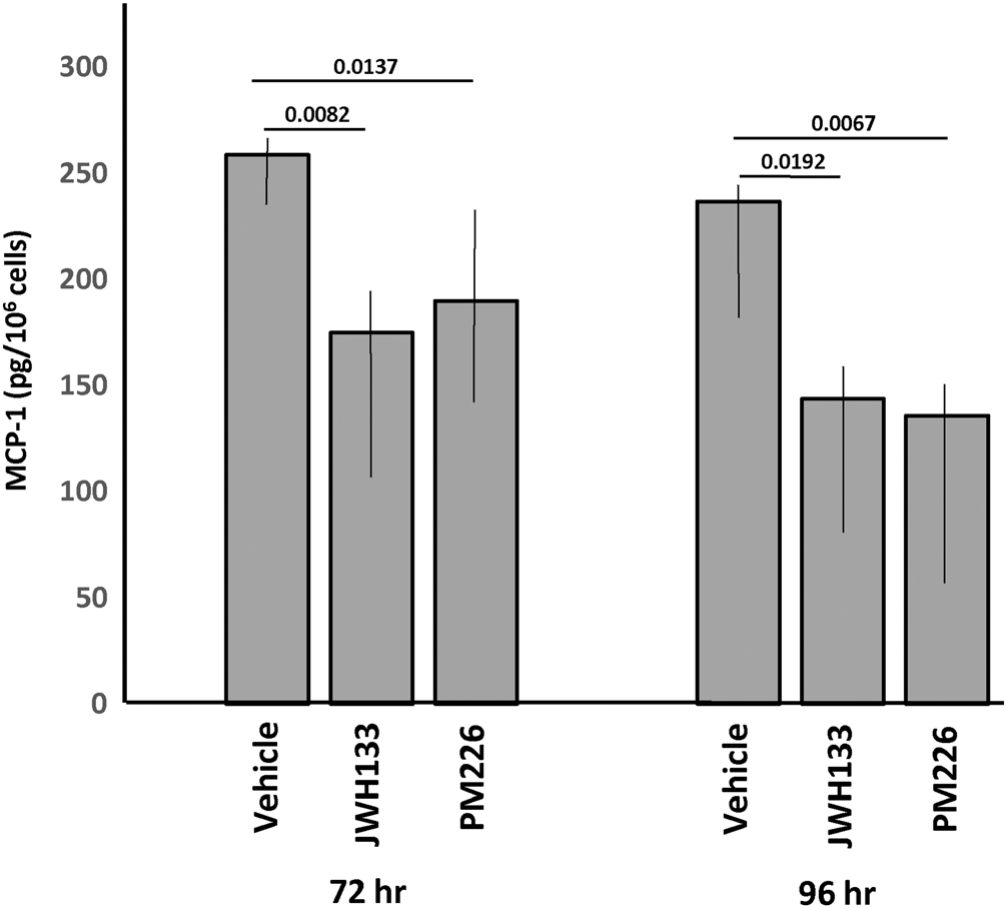
MCP‐1 levels in an mucopolysaccharidosis (MPS) IIIA patient cell line treated with CB2R agonists. Skin fibroblasts from a patient with MPS IIIA were grown to ~70% confluency and CB2R agonists JWH133 or PM226 were added to the culture media for 72 or 96 h. at a dose of 3 mM. Control cells were treated with vehicle (0.1% DMSO) alone. The levels of MCP‐1 released into the media were then measured by ELISA. Bar heights indicate the mean values from three independent experiments. *p* values are indicated.

**FIGURE 6:**
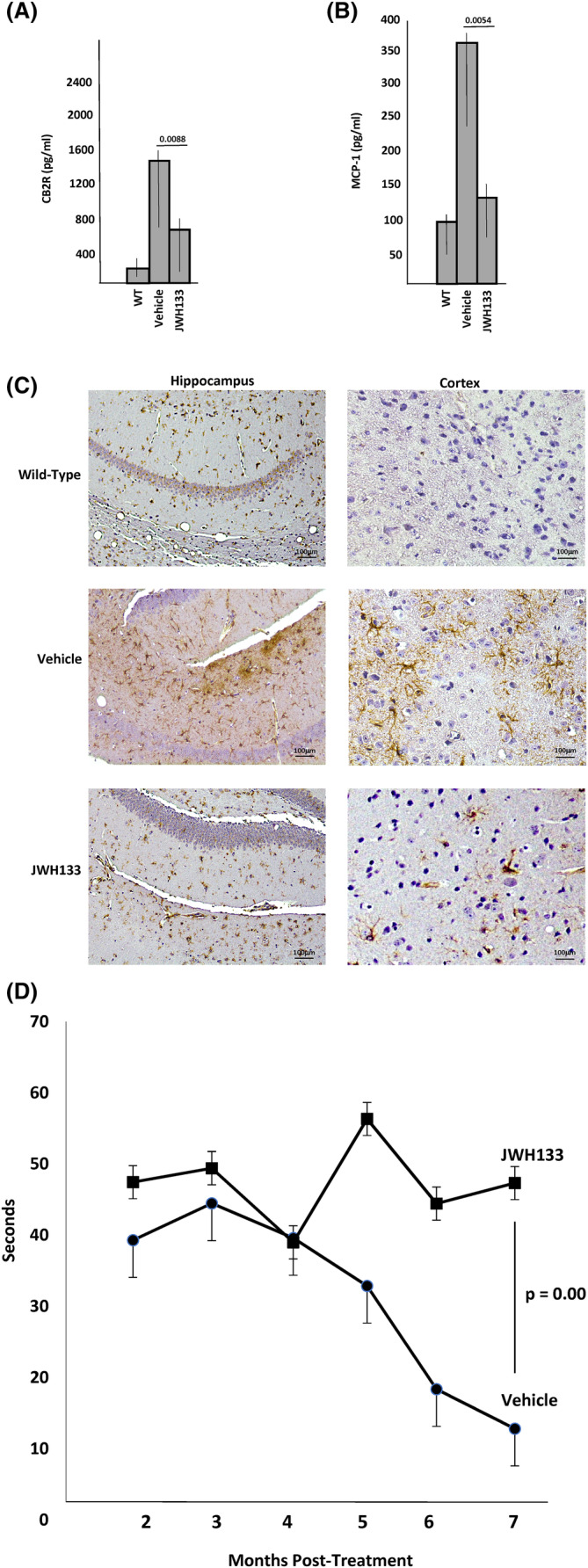
Treatment of mucopolysaccharidosis (MPS) IIIA mice with a CB2R agonist. Three‐month‐old MPS IIIA mice were treated once weekly with JWH133 (10 mg/kg) or vehicle (0.1% DMSO; *n* = 8/group) for 7 months, and (A) CB2R or (B) MCP‐1 levels were determined in the plasma by ELISA. *p* values are indicated. (C) Glial fibrillary acidic protein (GFAP), a marker of neuroinflammation, was detected in the hippocampus and cortex of vehicle or JWH133 treated MPS IIIA mice. Age‐matched WT mice from within the MPS IIIA colony were used as controls. Brown staining indicates GFAP. Note the reduced GFAP staining in the MPS IIIA mice treated with JWH133, similar to WT. (D) Starting at 2 months post‐treatment (5 months of age), the vehicle and JWH133 treated MPS IIIA mice were evaluated monthly by a hanging test analysis to assess motor strength and coordination. Note that the performance of the vehicle‐treated mice declined over the treatment period, while the JWH133 treated mice remained stable. The p value at 7 months post‐treatment is shown.

**FIGURE 7:**
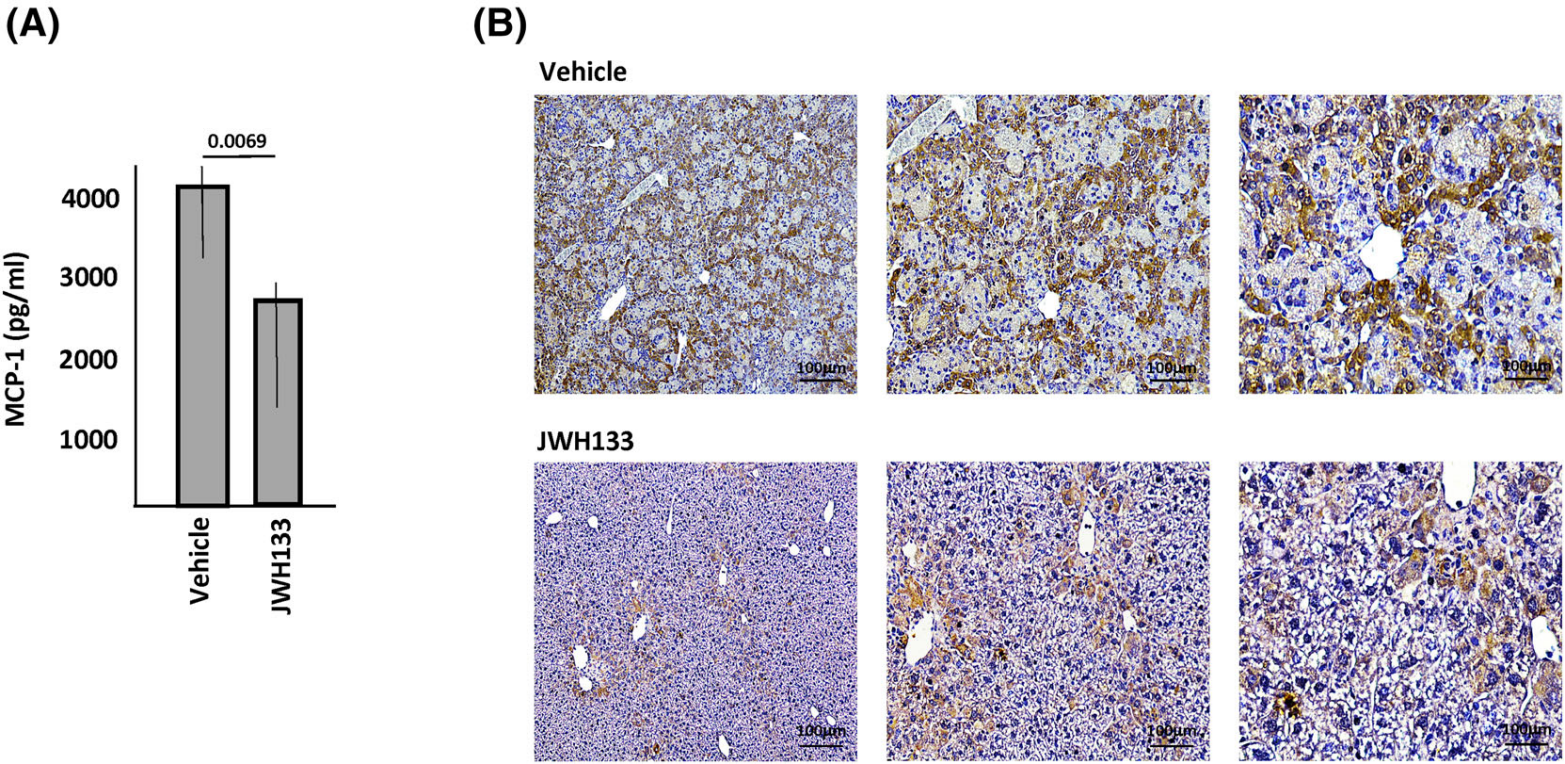
Treatment of Farber disease mice with a CB2R agonist. Two and one half‐month‐old Farber mice were treated with vehicle or JWH133 once weekly (10 mg/kg) for 6 weeks (*n* = 8/group) and (A) the levels of MCP‐1 were detected in plasma by ELISA or (B) in the liver by IHC. The *p* value is shown in (A) comparing vehicle and JWH133 treated mice (bar heights indicate the mean values from three independent experiments). Representative images are shown in (B). Brown staining indicates MCP‐1.

CB2R agonists have been used in many experimental disease models to reduce inflammation and/or modify disease progression, including models of neurologic disease.^13^, ^14^ Due to the lack of an existing treatment for MPS IIIA, we first evaluated this approach using a cell line from a MPS IIIA patient. As shown in Figure 5, treatment of these cells with two different CB2R agonists led to a reduction of MCP‐1 in the media, a chemotactic factor that is elevated in this and other LSDs.^24^, ^25^ We chose one of the agonists, JWH133,^26^, ^27^ to treat 3‐month‐old MPS IIIA mice (once weekly, 10 mg/kg) and found that at 7 months post‐treatment the plasma levels of CB2R and MCP‐1 were markedly reduced compared to mice treated with vehicle alone (Figure 6A,B). GFAP, a measure of neuroinflammation,^28^ also was reduced in the hippocampus and cortex of the MPS IIIA mice following treatment (Figure 6C), and motor function, which was evaluated by a hanging test, declined over time in mice treated with a vehicle but was stable in mice treated with JWH133 (Figure 6D). MCP‐1 levels were also reduced in plasma and liver from Farber disease mice treated with JWH133 (Figure 7).

## 4 DISCUSSION

For over three decades, the LSDs have been at the forefront of drug discovery for the rare diseases.^1^, ^2^, ^3^, ^4^ This has led to the approval of treatments for 14 of these diseases, including ERTs for 11 (Gaucher, Fabry, Pompe, MPS I, II, IV, VI, and VII, ASMD, alpha‐mannosidosis, and Batten), small molecule therapies for 4 (Gaucher, Fabry, Niemann‐Pick type C, and Cystinosis), and 1 gene therapy for Metachromatic Leukodystrophy. However, despite the remarkable successes in this field, major challenges remain. This includes the limited delivery and impact of many therapies at critical sites of pathology (including the muscle, bones, and brain) and the extremely high cost of treatment. In part, this latter challenge is due to the fact that most of these therapies are useful in only one disease, limiting their commercial potential.

Another emerging challenge is the ability to diagnose LSD patients prior to the onset of symptoms, including as newborns. Early diagnosis leads to improved care and also raises the possibility of early treatment intervention. However, at present it is not possible to reliably predict the clinical course in most LSD patients, particularly prior to clinical presentation. This has led many investigators to focus on identifying biochemical and other surrogate markers that can be easily measured in patients and are predictive of their clinical course.^7^, ^8^, ^9^, ^29^ In most cases these molecules are the disease‐specific substrates themselves (e.g., heparan sulfate for MPS III), but recent efforts have also focused on identifying molecules that represent downstream pathologic changes as well. Ideally, these changes might be occurring in more than one LSD. One example of such a molecule that shows promise for several neurologic LSDs is the neurofilament light chain.^30^

Similarly, recent efforts have also turned to identifying drugs that can be used in multiple LSDs. For example, Miglustat (marketed as Zavesca®) is a small molecule inhibitor of the enzyme glucosylceramide synthase and is used to treat patients with Niemann‐Pick disease type C and Gaucher disease.^31^, ^32^ Another example is N‐acetyl‐leucine, a small molecule that targets common neurologic mechanisms including neuroinflammation, neuronal membrane potential, and regulation of calcium channels. It is a recently approved treatment for Niemann‐Pick type C and under development for GM_1_ and GM_2_ gangliosidoses.^33^, ^34^ In principle, developing drugs such as these that target more than one disease should drive down the development costs and ultimately the cost of treatment for patients.

In this regard, we and others have focused on inflammation as a common therapeutic target in the LSDs.^35^, ^36^, ^37^, ^38^, ^39^, ^40^ This common abnormality, which has been attributed in part to activation of the NF‐kappa beta signaling pathway, has been documented in many LSD animal models, as well as in patients and contributes to pathology in many organs, including the brain and other sites that have proven difficult to treat by traditional therapies. Several years ago, these observations led us to repurpose a known drug, pentosan polysulfate (PPS), for use in the MPS disorders, and its efficacy was demonstrated in mouse and dog models of MPS I, a rat model of MPS VI, and in mice with MPS IIIA, where the impact on neurologic disease was shown.^41^, ^42^, ^43^ This has led to clinical trials in MPS I, II, and VI patients and the ongoing development of this treatment for multiple MPS disorders.^44^, ^45^, ^46^

As part of our effort to study inflammation in the LSDs, we have recently turned to the endocannabinoid system, an evolutionally conserved signaling system that consists of two essential G‐protein coupled receptors, CB1R and CB2R, and several lipid mediators that are carefully regulated by a series of synthetic and degradative enzymes.^12^, ^13^, ^47^ CB1R is primarily expressed in neurons and was originally identified as the ligand responsible for the neurotropic effects of the CB, Δ‐9 tetrahydrocannabinol (THC), while CB2R is primarily expressed on immune cells where it plays an important role in modulation of the immune system.^12^, ^13^, ^48^, ^49^ Notably, CB2R expression is upregulated in many disorders associated with inflammation, and CB2R agonists have anti‐inflammatory properties that impact disease progression in many disease models.^14^, ^48^, ^49^ Since chronic inflammation is a common feature of many LSDs, independent of the individual enzyme deficiency or type of substrate that is accumulating, we hypothesized that CB2R expression may be elevated in the LSDs and that CB2R agonists may have beneficial effects in one or more of these disorders.

We initiated our work by evaluating the expression of CB2R in plasma from six LSD animal models, chosen randomly among the breeding colonies we maintain in our laboratory. Among these, we found a significant elevation of CB2R in Farber disease and MPS IIIA mice, followed by MPS I and Fabry disease mice. We did not find significant elevation in plasma from ASMD mice or rats with MPS VI. For this analysis, we purposely chose aged LSD animals that exhibited tissue pathology and/or clinical abnormalities, and compared them to age‐matched WT mice derived from within the colonies. To our knowledge, this is one of the only studies in which plasma CB2R levels have been analyzed and shown to correlate with disease. Since CB2R is a G‐coupled receptor generally found in the outer membrane of blood cells, it is not clear why it is elevated in the plasma. We hypothesize that it may be present in small membrane fragments derived from disrupted cells that remain in the plasma after centrifugation, or perhaps is associated with lipoproteins or other plasma components. However, this remains to be proven. Nonetheless, CB2R is clearly elevated in several LSDs and this likely reflects their activated immune state and the proliferation of blood‐derived immune cells that occurs in these disorders.

To confirm the plasma CB2R findings, we next performed IHC analysis on tissues from the two disease models (Farber and MPS IIIA) that exhibited the highest plasma expression. High CB2R levels were also found in the tissues, including the liver, spleen, and brain of Farber mice and the liver and brain of MPS IIIA mice. qPCR analysis of the MPS IIIA animals confirmed these findings and indicated that the IHC observations were due to elevated *Cb2r* gene expression. We also investigated whether the elevated CB2R expression in these animals was associated with the activated inflammatory state of these animals, as has been shown in other diseases, and would respond to treatments that are known to reduce inflammation. To this end, we treated the Farber mice by ERT using recombinant acid ceramidase. Farber disease is due to the inherited deficiency of acid ceramidase, resulting in the accumulation of the lipid ceramide, which leads to chronic inflammation, cell death, and organ damage.^50^ ERT has been shown to reduce ceramide levels, leading to the resolution of inflammation and improvement in organ pathology.^23^ Notably, we found that six‐week ERT treatment reduced the levels of CB2R in the Farber mice, consistent with its known impact on inflammation. In the future, it will be of interest to study other treatment modalities that reduce inflammation in this and other LSDs and evaluate their impact on CB2R expression as well.

We then evaluated the therapeutic potential of CB2R modulation in these diseases. CB2R agonists have been shown to reduce inflammation in many disease models, and some have been shown to cross the blood–brain barrier.^12^, ^48^, ^49^ We chose two of these agonists and evaluated them using skin fibroblasts obtained from an MPS IIIA patient, and found that both significantly reduced the release of MCP‐1 into the culture media. MCP‐1 is an inflammatory chemokine that is elevated in this and other LSDs.^24^, ^25^ Based on this finding, we chose one CB2R agonist (JWH133)^26^, ^27^ and evaluated it in vivo in the MPS IIIA mice. After 7 months of treatment (once weekly, 10 mg/kg), the treated 10‐month‐old MPS IIIA mice exhibited reduced CB2R and MCP‐1 expression in the plasma, reduced GFAP staining in the brain, and improved performance in a hanging test that accessed motor strength and coordination. This latter finding was due to maintenance of stable function in the treated mice, versus declining function in the MPS IIIA mice treated with vehicle alone. To confirm these findings in MPS IIIA, we also treated Farber mice with JWH133 and found reduced MCP‐1 in plasma and liver.

Overall, these findings revealed the potential of CB2R as a novel biomarker and therapeutic target for the LSDs. Importantly, it is elevated in several different LSD models, consistent with the chronic inflammation that is common among them, and at least in the case of MPS IIIA mice, it appears to follow the progression of the disease (e.g., was not elevated at 2 months of age but was highly elevated by 10 months, where the disease pathology is evident). It is not clear why CB2R was not elevated in plasma of ASMD and MPS VI animals, which also exhibit systemic inflammation, but perhaps the age of these animals (5 months for ASMD mice and 8 months for MPS VI rats) was not sufficient to activate this pathway in the blood. Further studies are needed to elucidate these findings and, importantly, determine if the findings in these animal models correlate with those in LSD patients.

The impact of the CB2R agonists in MPS IIIA and Farber disease mice is also worthy of further investigation. Consistent with its impact on other diseases, JWH133 reduced the levels of CB2R and the inflammatory cytokine, MCP‐1, and in the case of MPS IIIA also improved motor function. Notably, GFAP staining in the brain was also reduced, indicating the ability of this molecule to impact neuroinflammation. While these results provide important proof‐of‐concept for using CB2R agonists in these and perhaps other LSDs, studies that evaluate additional clinical and pathological endpoints in the individual models, including survival, are needed. Of note, CB2R activation has also been shown to reduce neuronal loss, protect the blood–brain barrier, elevate beneficial M2 microglia in other disease models, and regulate TFEB‐mediated autophagy, an abnormality that is evident in most LSDs.^50^, ^51^ It is therefore possible that CB2R treatment may have additional effects in the LSDs, including unexpected outcomes as we observed with PPS, where it was shown to reduce glycosaminoglycan storage in the MPS disorders in addition to reducing inflammation. Indeed, the cross‐talk between inflammation and these other pathways is an important subject for future investigations.

These studies extend our ongoing investigation of the endocannabinoid system in the LSDs.^47^ Previously, we found that CB1R expression was reduced in the brain of ASMKO mice, and that treatment of these mice with inhibitors of the enzyme fatty acid amide hydrolase, which activates this receptor, led to reduced sphingomyelin levels and improvements in tissue pathology and clinical findings.^52^ This followed early studies showing that treatment of astrocytes with THC led to activation of CB1R and the enzyme neutral sphingomyelinase, resulting in sphingomyelin degradation.^53^ Others have recently reported that aged astrocytes exhibit cholesterol storage in lysosomes that is due, at least in part, to reduced expression of the NPC1 protein that is deficient in Niemann‐Pick type C disease, and that this abnormality could be alleviated by treatment with CBs, including cannabidiol (CBD).^54^ Thus, the endocannabinoid system remains a potentially important target for the treatment of these and other LSDs and is worthy of further study.

In conclusion, the results reported here are the first investigation of CB2R in the LSDs and the first to reveal its potential as a biomarker and therapeutic target for these diseases. Future studies should extend these findings in the LSD animal models and correlate the animal results to humans.

## AUTHOR CONTRIBUTIONS

Calogera M. Simonaro and Edward H. Schuchman conceived of all experiments analyzed the data and wrote the paper. Makiko Yasuda provided plasma from the Fabry mice and reviewed the data and the paper.

## FUNDING INFORMATION

Funding for this study was provided by a grant from the Mucopolysaccharidosis Society to Calogera M. Simonaro (IF2669039) and gifts from the Wylder Nation (0285‐8019) and Genetic Disease Foundation (0285‐2690).

## CONFLICT OF INTEREST STATEMENT

Calogera M. Simonaro and Edward H. Schuchman are inventors on a patent describing the use of CB2R as a biomarker and/or therapeutic target for the LSDs. Makiko Yasuda has no competing interests.

## INFORMED CONSENT

This article does not contain any studies with human subjects performed by any of the authors.

## ETHICS STATEMENT

All institutional and national guidelines for the care and use of laboratory animals were followed. All experiments were performed at the Icahn School of Medicine under protocols (#'s 08‐0108, 98‐0089, 2013‐1407, 202000236 and 202100005) approved by the Institutional Animal Care and Use Committee (IACUC), and adhered to the NIH guidelines for the use of experimental animals.

## Data Availability

All of the data generated for this study are included in the article. Primary source data are available on request from the corresponding author.
